# Supplementary material for: Promoter-anchored chromatin interactions predicted from genetic analysis of epigenomic data
Source: Nat Commun. 2020 Apr 28;11:2061. doi: 10.1038/s41467-020-15587-0 (PMC7188843; doi:10.1038/s41467-020-15587-0)
Supplement: Supplementary file 6 — Description of Additional Supplementary Files [file 41467_2020_15587_MOESM6_ESM.pdf]

Title: Supplementary Data 1
Description: Identification of 226 PAIs showing opposite directions in brain and blood samples.

Title: Supplementary Data 2
Description: Identification of 601 PIDSs associated with 15 human complex traits or diseases.

Title: Supplementary Data 3
Description: Gene-set enrichment analysis of the gene targets of trait-associated PIDS data by FUMA.
