# Supplementary material for: Promoter-anchored chromatin interactions predicted from genetic analysis of epigenomic data
Source: Nat Commun. 2020 Apr 28;11:2061. doi: 10.1038/s41467-020-15587-0 (PMC7188843; doi:10.1038/s41467-020-15587-0)
Supplement: Supplementary file 7 — Reporting Summary [file 41467_2020_15587_MOESM7_ESM.pdf]

nature research

Corresponding author(s): Jian Yang

Last updated by author(s): Mar 12, 2020

Reporting Summary

Nature Research wishes to improve the reproducibility of the work that we publish. This form provides structure for consistency and transparency in reporting. For further information on Nature Research policies, see Authors & Referees and the Editorial Policy Checklist .

Statistics

For all statistical analyses, confirm that the following items are present in the figure legend, table legend, main text, or Methods section.

n/a	Confirmed	
☐	☒	The exact sample size (*n*) for each experimental group/condition, given as a discrete number and unit of measurement
☒	☐	A statement on whether measurements were taken from distinct samples or whether the same sample was measured repeatedly
☐	☒	The statistical test(s) used AND whether they are one- or two-sided<br>*Only common tests should be described solely by name; describe more complex techniques in the Methods section.*
☒	☐	A description of all covariates tested
☐	☒	A description of any assumptions or corrections, such as tests of normality and adjustment for multiple comparisons
☐	☒	A full description of the statistical parameters including central tendency (e.g. means) or other basic estimates (e.g. regression coefficient) AND variation (e.g. standard deviation) or associated estimates of uncertainty (e.g. confidence intervals)
☐	☒	For null hypothesis testing, the test statistic (e.g. *F*, *t*, *r*) with confidence intervals, effect sizes, degrees of freedom and *P* value noted<br>*Give P values as exact values whenever suitable.*
☒	☐	For Bayesian analysis, information on the choice of priors and Markov chain Monte Carlo settings
☒	☐	For hierarchical and complex designs, identification of the appropriate level for tests and full reporting of outcomes
☐	☒	Estimates of effect sizes (e.g. Cohen's *d*, Pearson's *r*), indicating how they were calculated

Our web collection on statistics for biologists contains articles on many of the points above.

Software and code

Policy information about availability of computer code

Data collection	The peripheral blood mQTL summary data were from the Brisbane Systems Genetics Study (BSGS) (n=614) and Lothian Birth Cohorts (LBC) of 1921 and 1936 (n=1,366). The detailed description of data collection can be found in the McRae et al. 2018 Scientific Reports. We used the SMR software tool (version 1.03) to perform a meta analysis of the two cohorts.
Data analysis	We used the SMR software tool (version 1.03; https://cnsgenomics.com/software/smr/#Overview) to predict the promoter-anchored chromatin interactions (PAIs). All the analysis scripts are public available at https://github.com/wuyangf7/PAI.

For manuscripts utilizing custom algorithms or software that are central to the research but not yet described in published literature, software must be made available to editors/reviewers. We strongly encourage code deposition in a community repository (e.g. GitHub). See the Nature Research guidelines for submitting code & software for further information.

Data

Policy information about availability of data

All manuscripts must include a data availability statement. This statement should provide the following information, where applicable:

- Accession codes, unique identifiers, or web links for publicly available datasets
- A list of figures that have associated raw data
- A description of any restrictions on data availability

The summary statistics of the predicted PAIs are publicly available at http://cnsgenomics.com/shiny/M2Mdb/.
The summary statistics of the blood mQTL data are available at https://cnsgenomics.com/software/smr/#DataResource.
The summary statistics of the brain mQTL data are available at http://mostafavilab.stat.ubc.ca/xQTLServe/
The epigenomic annotation data are publicly available from the REMC (http://compbio.mit.edu/roadmap/).
The gene expression data of the GTEx project are publicly available at https://www.gtexportal.org/home/.
The Hi-C loops and TADs are available at https://www.ncbi.nlm.nih.gov/geo/query/acc.cgi?acc=GSE63525.
The PCHi-C loops and TADs are available at https://osf.io/u8tzp/.

The ChIA-PET loops and ChIP-Seq data are available from the ENCODE (https://www.encodeproject.org/experiments/).
The summary level ATAC-seq data are available at https://zenodo.org/record/1405945#.Xd4SbpMzaB0
The HRS data are available from dbGap (accession number phs000428.v1.p1) and the UK10K whole genome sequencing data are available at the European Genome-phenome Archive (accession number EGAS00001000108).

Field-specific reporting

Please select the one below that is the best fit for your research. If you are not sure, read the appropriate sections before making your selection.

- [x] Life sciences
- [ ] Behavioural & social sciences
- [ ] Ecological, evolutionary & environmental sciences

For a reference copy of the document with all sections, see nature.com/documents/nr-reporting-summary-flat.pdf

Life sciences study design

All studies must disclose on these points even when the disclosure is negative.

Sample size
Sample sizes in Lothian Birth Cohort (LBC) and Brisbane Systems Genetics Study (BSGS) corhorts were determined by the number of samples available for both genotype data and DNA methylation profiling, as described in the McRae et al. 2018 Scientific Reports. As far as we know, the sample size in the meta-analyzed mQTL data (n=1,960) is much larger than any previously published mQTL data sets.

Data exclusions
Since most of the GWAS data were from European ancestry, we restricted our mQTL analysis with only European individuals. Similar to previous studies, SNPs with minor allele frequency < 0.01 and DNA methylation probes in the major histocompatibility complex region were excluded.

Replication
We replicated our predicted PAIs in blood using an independent brain mQTL data. The result showed that 26.5% of blood PAIs were replicated in brain at a Bonferroni adjusted p-value threshold of 4.51×10e-06 and 66.31% were replicated at a nominal p-value threshold of 0.05.

Randomization
Randomization was not relevant to the study, because only summary level data was used for our analysis.

Blinding
We did not use blinding because this wasn't appropriate for the analyses performed in the study.

Reporting for specific materials, systems and methods

We require information from authors about some types of materials, experimental systems and methods used in many studies. Here, indicate whether each material, system or method listed is relevant to your study. If you are not sure if a list item applies to your research, read the appropriate section before selecting a response.

Materials & experimental systems

n/a	Involved in the study
[x]	[ ] Antibodies
[x]	[ ] Eukaryotic cell lines
[x]	[ ] Palaeontology
[x]	[ ] Animals and other organisms
[ ]	[x] Human research participants
[x]	[ ] Clinical data

Methods

n/a	Involved in the study
[x]	[ ] ChIP-seq
[x]	[ ] Flow cytometry
[x]	[ ] MRI-based neuroimaging

Human research participants

Policy information about studies involving human research participants

Population characteristics
All the samples were of European descent. The BSGS data set was measured on adolescent twins, and LBC data set was measured on subjects at an average age of 79 years. The DNA methylation level for each Individual probe was normalized after correcting for sex and age.

Recruitment
The BSGS data set (n=614) was from 177 families of European descent recruited as part of a study on adolescent twins. Families consist of adolescent monozygotic (MZ) and dizygotic (DZ) twins, their siblings, and their parents.
The LBC data set consists of the LBC1921 and the LBC1936 recruited by the Lothian Research Ethics Committee. Both LBC1921 and LBC1936 are longitudinal studies of ageing, with a focus on cognition, in groups of initially healthy older people. Of 1,366 individuals in LBC, 446 were from LBC1921 at an average age of 79 years, and 920 were from LBC1936 at an average age of 70 years.

Ethics oversight
This study is approved by the University of Queensland Human Research Ethics Committee (approval number: 2011001173).

Note that full information on the approval of the study protocol must also be provided in the manuscript.
